# T1 relaxation time is prolonged in healthy aging: a whole brain study

**DOI:** 10.55730/1300-0144.5630

**Published:** 2023-01-07

**Authors:** Hayriye AKTAŞ DİNÇER, Ahmet Muhteşem AĞILDERE, Didem GÖKÇAY

**Affiliations:** 1 Department of Biomedical Engineering, Institute of Natural and Applied Sciences, Middle East Technical University, Ankara, Turkey; 2 Department of Radiology, Faculty of Medicine, Başkent University Ankara, Turkey; 3 Department of Medical Informatics, Informatics Institute, Middle East Technical University, Ankara, Turkey

**Keywords:** T_1_ mapping, healthy aging, brain, quantitative MRI

## Abstract

**Background/aim:**

Measurement of tissue characteristics such as the longitudinal relaxation time (T1) provides complementary information to the volumetric and surface based structural analyses. We aimed to investigate T1 relaxation time characteristics in healthy aging via an exploratory design in the whole brain. The data processing pipeline was designed to minimize errors related to aging effects such as atrophy.

**Materials and methods:**

Sixty healthy participants underwent MRI scanning (28 F, 32 M, age range: 18–78, 30 young and 30 old) in November 2017–March 2018 at the Bilkent University UMRAM Center. Four images with varying flip angles with FLASH (fast low angle shot magnetic resonance imaging) sequence and a high-resolution structural image with MP-RAGE (Magnetization Prepared - RApid Gradient Echo) were acquired. T_1_ relaxation times of the entire brain were mapped by using the region of interest (ROI) based method on 134 brain areas in young and old populations.

**Results:**

T_1_ prolongation was observed in various subcortical (bilateral hippocampus, caudate and thalamus) and cortical brain structures (bilateral precentral gyrus, bilateral middle frontal gyrus, bilateral supplementary motor area (SMA), left middle occipital gyrus, bilateral postcentral gyrus and bilateral Heschl’s gyrus) as well as cerebellar regions (GM regions of cerebellum: bilateral cerebellum III, cerebellum IV V, cerebellum X, cerebellar vermis u 4 5, cerebellar vermis u 9 and WM cerebellar regions: left cerebellum IX, bilateral cerebellum X and cerebellar vermis u 4 5).

**Conclusion:**

T_1_ mapping provides a practical quantitative MRI (qMRI) methodology for studying the tissue characteristics in healthy aging. T_1_ values are significantly increased in the aging group among half of the studied ROIs (57 ROIs out of 134).

## 1. Introduction

According to the 2019 Revision of the World Population Prospects, by 2050, people over the age of 65 will be 16% of the total population [1]. Such a change in the pattern of population growth in favor of elderly people emphasizes the importance of aging studies in neuroimaging. During healthy aging, functional [2] and structural [3] changes occur in the brain. Beyond healthy aging, a notable amount of the aging population is susceptible to neurodegenerative diseases. The most common of them is Alzheimer’s disease, which affects approximately 30% of people aged 85 or older [4]. Understanding healthy brain aging is therefore crucial to be able to detect the early biomarkers of the age-related diseases.

Age-related structural brain changes including cortical thickness and signal intensity were reported in various areas [5]. These changes are so profound that age prediction from brain magnetic resonance (MR) images can feasibly be performed with machine learning [6] or based on the contrast at the tissue borders [7]. On the other hand, there are also age-related changes in the brain in intrinsic characteristics of the tissues. It is demonstrated that the main driver of the age-related gray matter (GM) volume loss in subcortical areas is the tissue property changes rather than atrophy [8]. In several studies, myelin, water content and iron were reported as the main factors that contribute to the structural changes in the aging brain. In MR imaging, these differences are observed through longitudinal relaxation time T_1_, the longitudinal relaxation rate R_1_ (R_1_ = 1/T_1_) and transverse relaxation time T_2_.

However, due to several technical limitations, the MRI signal bears inadequate information regarding the microstructure of the underlying tissues [9] through the T_1_, R_1_ and T_2_ measures. First, the signal is a nonlinear mix of several signal contrasts (T_1_, T_2_ or proton density (PD)). Second, choice of the data acquisition parameters, such as inversion time, echo time, flip angle, can only be determined suboptimally. Third, hardware artefacts in conventional MRI are difficult to circumvent through reverse engineering the signal. Due to these factors, use of conventional MRI techniques does not allow prediction of underlying properties of the human brain tissue. On the other hand, T_1_ relaxation time is a good indicator of local tissue density (such as water content), macromolecules (e.g., myelin), paramagnetic concentration (e.g., iron), as well as the lipid and protein compositions of the underlying tissue. Another superior property of the T_1_ is that it is less affected by hardware artefacts [10]. Additionally, T_1_ of the human brain has also been reported as a biomarker of development and maturation [11]. The abovementioned properties emphasize that T_1_ is a suitable parameter to investigate aging changes.

The studies that address quantification of T_1_ in the whole brain are scarce. In healthy aging, the studies that performed T_1_ mapping can be grouped based on the subject populations. Most of the studies conducted in this area are crosssectional designs. Steen et al. [12] examined age-related T_1_ changes in 18–72 years old 55 healthy volunteers and reported that T_1_ increased with age in the genu, frontal white matter, occipital white matter, putamen, and thalamus. Contrary to the subcortical areas, significant decrease in T_1_ with age was observed in cortical gray matter [12]. In a comprehensive study of 115 healthy subjects aged 4 to 72, it was reported that T_1_ exhibits a quadratic pattern in such a way that it begins decreasing in adolescence and reaches to minimum in 40–60 years but then starts to increasing [13]. Another study with 56 normal volunteers with ages 11–71 years reported a positive linear relationship between T_1_ and age in the genu of the corpus callosum (CC) and frontal white matter (WM) regions and a negative linear relationship in the left substantia nigra (SN) [14]. In line with the outcomes of Cho et al. (1997), the quadratic patterns were also shown in 3 frontoparietal WM regions and the right SN in this study [15]. In a recent study on 70 healthy subjects aged 20–76, a significant increase T_1_ in thalamus and WM but a decrease in amygdala, nucleus accumbens and the ventral-inferior putamen were reported [16]. Another recent study of 211 healthy participants aged 20–89 years found that there was negative linear relationship between T_1_ and age in putamen, thalamus and head of the caudate nucleus, but positive linear correlation in frontal lobe WM, globus pallidus and genu of the CC [17]. In all these crosssectional studies, the age range of the participants in the cohort seem to be a major confounding factor prohibiting comparison of the findings.

Despite the abundance of the crosssectional studies, there are only two longitudinal designs investigating age-related changes of T_1_. In a part of the LBC1936 longitudinal study (wave 2: N = 653, mean age = 73; wave 3: N = 442, mean age = 76) hippocampal T_1_ was demonstrated to significantly decrease across waves [18]. In the other longitudinal study (7-year period,17 healthy subjects, 51–77 years), an age related decrease of mean T_1_ in GM cortex and unchanged mean T_1_ in WM were reported [19]. As seen from the literature, there is no consistency in the reported T_1_ changes during aging and it seems that the limited number of ROIs targeted in these studies prohibit a general comparison across the findings.

In this study, we aimed to conduct an exploratory approach to understand T_1_ relaxation time differences in aging by studying the entire brain. The data processing pipeline was designed to minimize the errors related to aging effects such as atrophy. It is well known that age-related GM-WM loss and also brain atrophy become prominent after 70–80 years of life [20]. The age range of the old group participated in our study was chosen specifically to circumvent this problem.

## 2. Materials and methods

### 2.1. Participants

A total of 63 participants volunteered to participate in the study; however, 1 young and 2 old subjects were excluded from the study because of their claustrophobia. Hence, total of 60; 30 young (mean = 26.36 years, SD = 2.69, 12 F, 18 M) and 30 old (mean = 67.46 years, SD = 4.89, 16 F, 14 M) subjects participated in the study. Demographic information of the remaining 30 participants is given in Table. Classification of the participants into age groups was conducted according to the aging criterion of the United Nations [21].

The recruiting of the participants was conducted via distributed fliers, social media and with the help of our circle of acquaintances. All the participants signed informed consent according to the principles of the Declaration of Helsinki and the study was approved by Ankara University Clinical Research Ethical Committee. Exclusion criteria were history of a neuropsychological/psychiatric disorder, alcoholism, use of medication affecting the central nervous system (CNS) and cognitive decline in the aging group. None of the participants reported having a neuropsychological/psychiatric disorder or suffering from alcoholism and all of them stated that they were not using any medication affecting CNS. In addition, the participants included in the study did not have any physical disability preventing the application of the cognitive tests (visual, hearing, etc.), nor a metal prosthesis or pacemaker, and claustrophobia which would endanger their MR scanning. To determine whether the older participants were cognitively healthy, standardized mini mental state examination (SMMSE) [22] and geriatric depression scale (GDS) [23] were administered. The cutoff scores were 25 and 11, respectively. All participants fulfilled these criteria, as indicated in Table.

### 2.2 Procedure

Structural MRI data were collected at the National Magnetic Resonance Research Center (UMRAM) in Bilkent University, using a 3T MRI system (Siemens Magnetom Trio, Germany) in November 2017–March 2018. The scan time was 20 min. High resolution 3D anatomical brain images were collected with of MP-RAGE (Magnetization Prepared - RApid Gradient Echo) protocol (TR = 2500 ms, TE = 3.16 ms, bandwidth = 199 Hz/pixel, matrix 256 × 256, slice thickness 1mm, 256 slices, FOV = 256 × 256 (axial), number of averages = 1). Then 4 brain images with four different flip angles (3°, 5°, 15°, 30°) that adhered to the same imaging coordinates with the MPRAGE sequence were collected with FLASH (fast low angle shot magnetic resonance imaging) sequence (TR = 20 ms, TE = 4.15 ms, bandwidth = 199 Hz/pixel, matrix 256 × 256, with slice thickness 3 mm, 44 slices, FOV = 256 × 256 (axial), number of averages = 1). MPRAGE and FLASH sequences were preferred because MPRAGE yields high resolution images with low specific absorption rate (SAR) even in high magnetic field MRIs, and FLASH sequence introduces images varied contrast images in short scanning times. Moreover, these sequences are widely available in various scanners.

### 2.3 Data processing

#### 2.3.1 Preprocessing

Data collection is prone to head motion due to the long scan time. To remove head motion, images were deobliqued using AFNI’s 3dWarp program [24]. In addition, skull removal and bias field correction was performed with FAST tool [25]. For standardization, the images were aligned to the standard stereotaxic space (TLRC) by auto_tlrc program of AFNI [24].

#### 2.3.2. T_1_ mapping

It was previously reported that variable flip angle (VFA) provides a better alternative to conventional methods through improved precision and speed [26]. Hence, we used VFA with 4 different flip angles (FA) for mapping the spin lattice relaxation time (T_1_) of each voxel. FLASH is an appropriate sequence providing opportunity to collect images at different contrasts [27], allowing enhancement of data through different flip angles. For this purpose, we collected four FLASH images with varying flip angles (3°, 5°, 15°, 30°).

Eq. 1 demonstrates the intensity value I (x, y, z) of the voxel at (x, y, z) coordinates acquired with FLASH sequence. The intensity is calculated by tissue characteristics such as magnetization transfer constant (M_0_), longitudinal relaxation time (T_1_), transverse relaxation time (T_2_) and scanning parameters such as repetition time (TR), echo time (TE) and flip angle (α).


Eq (1) $${\text{I}}_{\alpha }(\text{x},\text{y},\text{z})=\frac{{\text{M}}_{0}(\text{x},\text{y},\text{z})\;{\text{e}}^{-\text{TE}/{\text{T}}_{2}*}\text{sin}(\alpha )(1-{\text{e}}^{-\text{TR}/{\text{T}}_{1}}))}{\left(1-\text{cos}(\alpha )\;{\text{e}}^{-\text{TR}/{\text{T}}_{1}}\right)}$$

Based on the requirement of VFA method, we collected four images with different flip angles. For really small flip angles (e.g., α = 3°) cos(α) approaches to 1; thus, the equation (1) is reduced to Eq. 2 [28]:


Eq (2) $${\text{I}}_{\alpha }(\text{x},\text{y},\text{z})={\text{M}}_{0}(\text{x},\text{y},\text{z})\;{\text{e}}^{-\text{TE}/{\text{T}}_{2}*}\;\text{sin}(\alpha )$$

In this way, the intensity value of the FLASH image collected with 3° flip angle is described as a constant c = M_0_(x, y, z)sin (3°˚). Hence, Equation 1 can be rewritten as follows:


Eq (3) $${\text{I}}_{\alpha }(\text{x},\text{y},\text{z})=\frac{\text{c}(\text{sin}(\alpha )/\text{sin}(3))\;(1-{\text{e}}^{-\text{TR}/{\text{T}}_{1}}))}{\left(1-\text{cos}(\alpha )\;{\text{e}}^{-\text{TR}/{\text{T}}_{1}}\right)}$$

I_α_ (x, y, z) in Equation (3) represents the intensity value observed in FLASH images acquired with 5°, 15°, and 30° flip angles, respectively, where c is acquired from the image with α = 3°. So far, all the parameters in Eq. (3) are known except for T_1_. The usage of 3 equations derived from 3 images and the only one unknown parameter (T_1_) make this problem overdetermined. Based on previous studies, the range of the T_1_ is determined as 0–4000 ms. For all these candidate values of T_1_, intensity I_α_(x, y, z) is calculated for all three images (α = 5°, 15°, and 30°) based on Eq. (3). Then, computed theoretical I_α_ for each T_1_ and measured real I_α_ in image is subtracted and squared for the voxel (x, y, z). Through a least square error fit, the T_1_ value of the I_α_ which has the smallest error is assigned as the T_1_ value of that particular voxel. This method is implemented in MATLAB [29] to create 3D T_1_ maps of each participant.

#### 2.3.3. Segmentation

**Figure 1:**
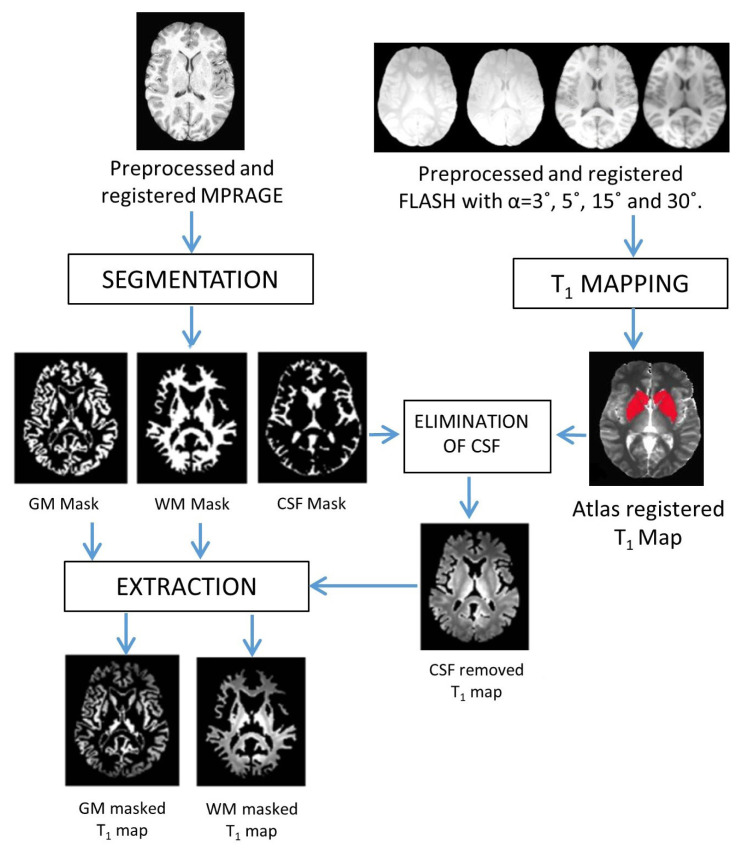
Data processing pipeline for T_1_ mapping (red: bilateral caudate and putamen ROIs from the atlas).

FSL’s FAST tool [25] is used to segment the MPRAGE image, into three tissue types: WM, GM, and cerebrospinal fluid (CSF). The MPRAGE image is aligned to the T_1_ map image through the FLASH images. Hence, the binary CSF mask produced from the MPRAGE image can be used to remove the CSF from the T_1_ maps, to take care of the data processing errors due to the atrophy in the cortex and ventricular enlargement in the subcortical areas of the aging group. The binary masks of WM and GM produced from the MPRAGE are then used as a basis for T_1_ measurements in specific ROIs depicted by the atlas (see Figure 1). The usage of WM masks is especially important to minimize the partial volume effects (PVE) in the aging brain. These procedures are illustrated in Figure 1.

#### 2.3.3. ROI analysis

AFNI’s CA_N27_ML atlas is used for ROI signal measurements. We have created ROIs based on this atlas and measured average T_1_ values of each participant on a total of 134 regions: 12 regions from subcortical area, 72 regions for cortex and 50 regions in cerebellum (25 for each GM and WM area). Additionally, 72 WM masks in cortex are created for elimination of WM tissues surrounding gyri.

#### 2.3.5. Statistical analysis

The data were analyzed using SPSS (version 17.0) statistical software. Kolmogorov-Smirnov test is applied for T_1_ values of each ROI to determine the distribution of the data. Then, according to the outcome either independent samples t-test or Mann-Whitney U test is conducted to investigate the T_1_ variations of the defined ROIs between young and old participants. For each ROI, Bonferroni correction is done separately to account for the multiple measurements of the same ROI from both hemispheres (n = 2). The false positive rate is controlled using family-wise error (FWE) correction for multiple comparisons, and thresholded at p < 0.05 at the cluster level.

## 3. Results

### 3.1 Subcortical area

**Figure 2:**
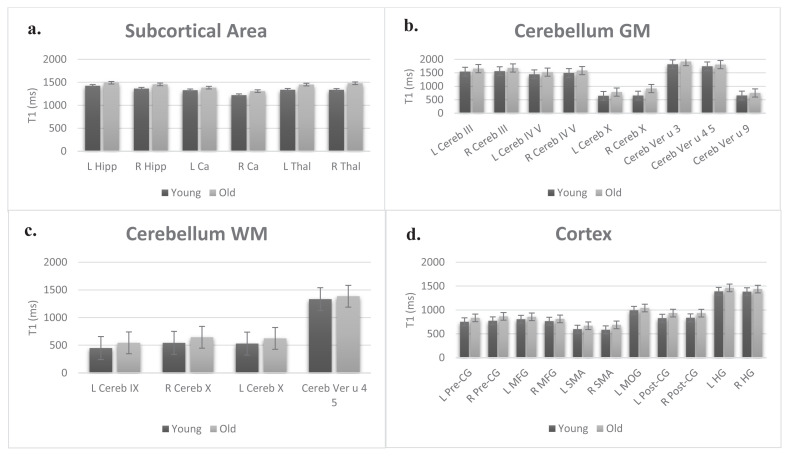
Average T_1_ values measured in subcortical area (a), cerebellum GM (b), cerebellum WM (c) and cortex (d). (R: right; L: left; u: unilateral; Hipp: hippocampus; Ca: caudate; Thal: thalamus; Cereb: cerebellum/cerebellar; Pre-CG: precentral gyrus; MFG: middle frontal gyrus; SMA: supplementary motor area; MOG: middle occipital gyrus; Post-CG: postcentral gyrus; HG: Heschl’s gyrus.

A Kolmogorov-Smirnov test indicated that T_1_ values in this region had a normal distribution (p ≥ 0.05). Independent Samples t-test demonstrated that average T_1_ value in bilateral hippocampus, caudate and thalamus of the old group were significantly higher than those of younger counterparts. Figure 2a shows the average T_1_ values that significantly increased in the aged group.

### 3.2. Cerebellum

Kolmogorov-Smirnov test indicated that the T1 values in GM regions of the cerebellum were not normally distributed (p ≤ 0.05). A Mann-Whitney U test indicated significant age-related differences in average T_1_ measurements in 9 GM regions of the cerebellum. The average T_1_ values of old participants measured in bilateral cerebellum III, cerebellum IV V, cerebellum X, cerebellar vermis u 4 5 and cerebellar vermis u 9 were significantly higher than those of the young group as seen in Figure 2b. Similarly, a Kolmogorov-Smirnov test indicated that T_1_ values measured in WM regions in the cerebellum did not have a normal distribution (p ≤ 0.05). The results of the Mann-Whitney U test showed that the average T_1_ value measured in left cerebellum IX, bilateral cerebellum X and cerebellar vermis u 4 5 of the old participants were significantly higher than those of young participants (Figure 2c).

### 3.3. Cortex

A Kolmogorov-Smirnov test demonstrated that T_1_ values in this area violated the normality assumption (p ≤ 0.05). According to the Mann-Whitney U test, age-related significant increase in average T_1_ values were reported in 38 ROIs including bilateral precentral gyrus, bilateral middle frontal gyrus, bilateral supplementary motor area (SMA), left middle occipital gyrus, bilateral postcentral gyrus bilateral and bilateral Heschl’s gyrus. The average T_1_ values measured in these ROIs are given in Figure 2c.

Overall, we have demonstrated that there is a trend indicating that average T_1_ values measured in the brain significantly prolonged with increasing age. We did not observe a significant reduction of T_1_ in any of the ROIs.

## 4. Discussion and Conclusion

Quantitative MRI techniques with different contrast mechanisms are used in the clinic to provide valuable information about both normal and pathological brain tissues. There are several studies reporting a relationship between myelin and T_1_ [30, 31]. Reduced myelination of the underlying tissue tends to prolong T_1_ [32]. Rooney et al. (2007) showed the relative contribution of iron content to R_1_ maps [33]. Higher iron concentration in deep brain nuclei [34] and cortex [35] reduces T_1_. In addition, T_1_ is linearly proportional to the water content [36] and age-related reduced water content tends to reduce T_1_ [35]. Furthermore, it is important to consider that these factors are coupled with each other. While myelin is a key factor determining the interpretation of T_1_, the water content of the myelin is a confounding factor.

Previous studies reported differences of T_1_ in subcortical and cortical areas of patients with Parkinson’s [37] and Alzheimer’s disease [38]. These outcomes indicate the potential of T_1_ mapping in neurodegenerative disease research. Determination of T_1_ variation in the brain is also important from a cytoarchitectural point of view. It was shown that R_1_ maps of the visual cortex and retinotopic maps are associated with many visual area borders [39].

In our study, T_1_ values in the whole brain of young and old participants were measured based on 134 ROIs from the CA_N27_ML atlas. This allowed us to investigate T_1_ changes during healthy aging comprehensively. To the best of our knowledge, this is the first study investigating age-related changes of T_1_ relaxation time in the ROIs selected from the whole brain using an exploratory approach. The major finding of this study is that age-related T_1_ prolongation is observed in various subcortical and cortical brain structures, as well as cerebellar regions. In a cohort of young (18–35 years old) and early aging (60–78 years old) groups, age-related prolongation of T_1_ in several subcortical, cortical, and cerebellar areas (57 of 134 ROIs) is observed. All the significant changes observed in this study are related to prolongation of T_1_. We did not observe a significant decrease in T_1_ in any of the ROIs between these two age groups.

In terms of subcortical structures: We observed significant T_1_ increases in bilateral caudate, thalamus and hippocampus in the older group relative to younger participants. The findings of our study in these subcortical areas replicated the outcomes of some previous studies indicating an age-related T_1_ increase in basal ganglia [40] and thalamus [12, 16, 17] in elderly. A possible origin of increased T_1_ in subcortical regions in our experiment might be explained by the concomitant loss of myelin in these structures. Age-related demyelination in thalamus [41] and in basal ganglia [42] have been previously reported. Although increased iron content in elderly shortens T_1_, a degeneration of the myelin sheets has been observed in elderly due to iron accumulation [43]. Water content also influences T_1_ and it is strongly correlated with iron in several regions including caudate and thalamus [34]. Unfortunately, the underlying region specific mechanism of the association among iron, myelin and T_1_ is poorly understood [34, 42]. Therefore, further studies with complementary techniques (such as MR spectroscopy) and multiparameter approaches investigating myelin water fraction (MWF) are needed to unveil the individual aging mechanisms.

Our outcomes contradict with some recent studies which reported a decreased T_1_ [16] and no change [44] in deep GM structures (e.g., basal ganglia) of old participants. This conflict might stem from the difference between the ROI measurements in these studies and ours. For instance, in one of these studies, manual drawing of the ROIs on a single midslice on the population-averaged map was used [16]. In our study 134 3D ROIs are created for each participant’s MR image by registering to the stereotaxic space and atlas. We developed an automated data processing pipeline free from biases stemming from subjective measurements. Therefore, the discrepancy between our results and others’ may be attributed to the choices in such quantification techniques. In the other study [44] presenting conflicting outcomes with ours, T_1_ was measured on an ROI which was a combination of several subcortical structures (e.g., caudate, putamen, thalamus, etc.). Such combination of structures might cause losing regionally-specific information which is important in aging [45]. Finally, the CSF T_1_ values are prominently different than those of GM and WM areas, deserving special treatment. We removed the CSF areas in subcortical and cortical regions to minimize the effect of age-related atrophy and ventricular enlargements on tissue concentrations and to measure T_1_ more precisely. We believe that some portion of the reduction of T_1_ reported in other studies might result from confounding factors that were not accommodated during the initial data processing steps.

In terms of cortical structures: Our findings of elongated T_1_ are in line with some studies on somatosensory cortex [46] and on occipital gyrus [47]. On the other hand, a decrease in T_1_ with aging [12, 44, 48, 49] or no change in cortex [50] were reported in other works. The difference between the age range of the participants in Gracien et al.’s study (2017) and ours possibly presents the main source of this contradiction (i.e. 55–71 years in the former, 18–78 years in the latter). Earlier, it has been reported that the T_1_ differences may follow a quadratic pattern [13]. Gracien’s study (2017) might be focusing on the decreasing range of this pattern while our study focuses on the increasing range. Global T_1_ measurements based on frequency distributions (or spectra) were conducted in two studies [48, 49]. In these studies, the aging effect was reported as decreased T_1_ in GM and increased T_1_ in WM. These studies did not distinguish the T_1_ changes with respect to ROI. Hence, a comparison between these studies and ours is impossible.

In terms of cerebellum, we found T_1_ prolongation in several structures including cerebellum III, X, and vermis. There are very few studies investigating T_1_ changes in aging in the cerebellar area. The sheet-like structure of cerebellum is composed of complex cellular layers which are really thin (≤1 mm) [51]. Moreover, the myelination degree [52] varies in different cortical depths, also cell sizes, distribution and densities across the cerebellar lobes [53]. Perhaps the complexity of the cerebellar anatomy is a prohibiting factor in ROI based analysis of T_1_ changes in cerebellum. We have replicated outcomes of Saito (2009) reporting an increase in WM anterior subsegment of cerebellum. On another front, one recent study by Badve et al. (2015) reported that age-related changes were nonexisting in cerebellum and cerebellar vermis [14]. In Badve and colleagues’ study, quantitative metrics were measured with magnetic resonance fingerprinting (MRF) technique, in which slice thickness was higher than that in our study. Resolution differences might have been effective in the conflicting outcomes between their results and ours.

Although there are abundant previous studies focusing on subcortical structures, the literature is not rich in evaluating the T_1_ variation in cortex and cerebellum. The complex and folded topology of cortex and cerebellum is a possible reason of the absence of qMRI and microstructural studies in these areas. There are several limitations imposed by the aging process and complexity of the automatic processing of brain images. The cortical thinning [5] is a crucial factor that would introduce PVE and worsens interpretation of the cortical data which already has a complex structure.

The present study has several limitations that should be addressed. The variable flip angle method which we have used in the present study is reported to overestimate T_1_ values in vivo due to imperfect spoiling and B_1_ bias [54]. Although the correction methods for flip angle inhomogeneities were proposed previously [55], many qMRI studies—including ours—lack a correction while computing the T_1_ maps. Only after flip angle corrections are done on individual brains, the T_1_ evaluation methods would indicate accurate changes between age groups. It is important to identify within which range flip angle inhomogeneities are tolerated to predict T_1_ maps. To test the restrictions of our study, we ran simulations to compute the range of errors in predicting the T_1_ values. We can safely state that our results are valid, if the variability of flip angles remain in the range of ±10% in comparison to the settings of the MR protocol (results available upon request).

This study has a crosssectional design which limits the interpretation individual aging patterns. Longitudinal studies should be conducted in the future to provide a better understanding of the age-related changes and minimize intersubject variations. To sum up, our report of increased T_1_ characteristics in healthy aging is centered on young and old adults and limits the generalization to ages outside this age range.

Furthermore, the interpretation of T_1_ measurements is affected by other factors such as the initial atlas registration and regional boundaries of the anatomical structures. Another limitation worthy to note is the method of registration. There are two different age groups whose anatomical properties differ a lot. We have created special masks for each participant to remove CSF for a better differentiation of WM and GM. This way, at least we were able to remove the age-dependent increase in ventricles and the effects of atrophy. Age-specific atlases may be used for future studies. In addition, when we consider biological variation introduced by the regional heterogeneity of the aging patterns, the complexity of predicting MR signal characteristics represented by T_1_ should be considered carefully.

## Figures and Tables

**Table:** The demographical information of the subjects.

	Old	Young
Age (mean ± SD)	67.46 ± 4.89	26.36 ± 2.69
Sex	Female = 16	Female = 12
	Male = 14	Male = 18
SMMSE (mean ± SD)	26.34 ± 0.46	-
GDS (mean ± SD)	1.28 0.84	-
